# Cell Counting and Cell Cycle Analysis of Simple Non-Cultured Endothelial Cell Injection (SNEC-I) Therapy: Characterization for Clinical Translation

**DOI:** 10.3390/cells14130986

**Published:** 2025-06-27

**Authors:** Darren S. J. Ting, Gary S. L. Peh, Dawn J. H. Neo, Xiao Yu Ng, Belinda Y. L. Tan, Raymond C. B. Wong, Hon Shing Ong, Jodhbir S. Mehta

**Affiliations:** 1 Tissue Engineering and Cell Therapy Group, Singapore Eye Research Institute, Singapore 169856, Singapore; d.s.j.ting@bham.ac.uk (D.S.J.T.); garypeh.seri@gmail.com (G.S.L.P.); dawnnjh.seri@gmail.com (D.J.H.N.); nxiaoyu87@gmail.com (X.Y.N.); ylb.tan@gmail.com (B.Y.L.T.); honshing@gmail.com (H.S.O.); 2 Ophthalmology & Visual Sciences Academic Clinical Programme (EYE-ACP), Duke-NUS Graduate Medical School, Singapore 169857, Singapore; 3 Academic Unit of Ophthalmology, Department of Inflammation and Ageing, College of Medicine and Health, University of Birmingham, Birmingham B15 2TT, UK; 4 Birmingham and Midland Eye Centre, Sandwell and West Birmingham NHS Trust, Birmingham B18 7QH, UK; 5 Academic Ophthalmology, School of Medicine, University of Nottingham, Nottingham NG7 2RD, UK; 6 Centre for Eye Research Australia, Royal Victorian Eye and Ear Hospital, East Melbourne, VIC 3002, Australia; ching.wong@unimelb.edu.au; 7 Corneal & External Eye Disease, Cataract & Comprehensive Ophthalmology, Refractive Surgery, Singapore National Eye Centre, Singapore 168751, Singapore

**Keywords:** bullous keratopathy, cell therapy, corneal transplant, corneal endothelial cell, corneal endothelial disease, Fuchs corneal endothelial dystrophy, keratoplasty, regenerative medicine

## Abstract

Human corneal endothelial cell therapy has recently emerged as a novel solution to treat corneal endothelial diseases. We previously demonstrated the potential of utilizing non-cultured primary corneal endothelial cells (CEnCs) isolated from donor corneas with low endothelial cell density for simple non-cultured endothelial cell injection (SNEC-I) therapy. This study aimed to develop a robust and semi-automated approach for cell counting, characterize the extent of cellular manipulation, and evaluate the translational workflow. To address this, we evaluated manual and automated cell counting approaches and characterized the extent of manipulation of CEnCs through the analysis of cell cycle status, gene expressions, and transcriptomic profiles with single-cell RNA-sequencing. The translational feasibility and functionality of SNEC-I therapy were examined using an established rabbit model of bullous keratopathy. Manual hemocytometry and automated cell-counters exhibited comparable accuracy and reproducibility. Analysis of cell cycle status, cell cycle genes (*n* = 11), and transcriptomic profiles revealed close resemblance between the native corneal endothelium and its donor-matched SNEC-I-harvested cells. Successful resolution of bullous keratoplasty in the pre-clinical model supports the feasibility, efficacy, and safety of SNEC-I therapy. In conclusion, SNEC-I therapy serves as an attractive corneal endothelial therapeutic approach (from a regulatory standpoint) in view of the minimal extent of cellular manipulation.

## 1. Introduction

A functional corneal endothelium is critical for the transparency and deturgescence of the cornea. With a starting density of over 3000 cells/mm^2^ at birth, the corneal endothelial cell density (ECD) gradually diminishes at a physiological rate of 0.6% per year, with approximately 2000 cells/mm^2^ remaining over the age of 70 years [1,2]. In healthy eyes, this physiological reserve is generally sufficient beyond the functional threshold of the corneal endothelium to maintain corneal transparency. Nonetheless, a wide array of causes, including genetic, infective, and traumatic causes, can accelerate the loss of the corneal endothelial cells (CEnCs), leading to visual impairment and blindness [3].

Fuchs endothelial corneal dystrophy (FECD) and pseudophakic bullous keratopathy are two of the most common causes of corneal endothelial diseases worldwide [3,4]. They have been consistently shown as the leading indications for corneal transplantation worldwide [4,5,6]. Currently, there is a significant shortage of suitable donor corneas worldwide, with approximately 1 in 70 patients receiving a donor cornea for corneal transplantation [7]. To overcome this issue, various strategies and innovations have been implemented to improve the eye donation rate and surgical techniques (hence a better graft survival outcome) and reduce the dependence on donor corneas for corneal transplantation (e.g., endothelial cell therapy) [3,8,9,10,11].

With improved surgical techniques and understanding of the corneal anatomy and immunology, the field of keratoplasty has observed a paradigm shift from penetrating keratoplasty to lamellar keratoplasty for selectively replacing the diseased anterior and posterior corneas [3,5,12,13]. Such innovation not only helps improve the visual outcome and graft survival rate [14] but also increases the utilization of donor corneas [15,16]. For instance, donor corneas that have inadequate ECD can be used for anterior lamellar keratoplasty [14]. Nonetheless, the global shortage of donor corneas remains a persistent barrier to corneal transplantation.

The cells of the human corneal endothelium are known to have a very limited capacity to proliferate in vivo as they are locked within the G1 phase of the cell cycle [17,18,19]. However, over the past decade, various research groups have demonstrated the potential of endothelial cell therapy in treating corneal endothelial disease, serving as an exciting therapeutic avenue in reducing the need for donor corneas [11,20,21]. Numa et al. [22] recently reported favorable long-term efficacy and safety of corneal endothelial cell injection therapy in 11 patients. Normal corneal endothelial function was restored and maintained in 10 of the 11 eyes, with significant visual improvement lasting up to 5 years post-injection therapy. Peh et al. similarly demonstrated the potential of corneal endothelial replacement therapy, delivered via a cell injection approach or on a tissue-engineered scaffold, in restoring the corneal endothelial function in pre-clinical rabbit models [20,21]. However, the translation of corneal endothelial cell-based therapy from bench to bedside using culture-expanded cells requires specialized expertise, equipment, and facilities that are compliant with Good Manufacturing Practice (GMP), posing significant translational and regulatory barriers [23].

Our group recently explored and reported the feasibility and efficacy of simple non-cultured endothelial cell injection (SNEC-I) therapy in treating corneal endothelial disease in a pre-clinical rabbit model of bullous keratopathy [24]. This approach utilizes donor corneas that are generally considered unsuitable for conventional endothelial keratoplasty (e.g., ECD of <2200 cells/mm^2^) by harvesting the functional CEnCs from these corneas and pooling these cells into a sufficient concentration (without involving any form of cellular expansion), followed by injecting the cells into the anterior chamber [24]. More importantly, this alleviates the issue of donor tissue wastage and maximizes the usage of retrieved donor corneal tissues with lower ECD that may otherwise be discarded.

However, there remain several important questions to be answered prior to the translation of the proposed SNEC-I therapy to the clinic. It is critical for clinicians and the regulatory body to be able to reliably determine the number of harvested CEnCs prior to injection so that the desired outcome can be consistently achieved. In addition, while SNEC-I therapy obviates the need for cellular propagation (as opposed to the conventional cultured corneal endothelial cell therapy approach), the extent of “cellular manipulation” has yet to be determined. This has important regulatory implications, as tissues or cells requiring a lesser degree of manipulation, when compared with conventional processes involved in cellular expansion, could potentially be used to navigate a more rapid regulatory approval process [23]. In view of these highlighted issues, this study aimed to (1) develop a robust approach in obtaining the overall cellular yield using a semi-automated cell counter; (2) characterize the cell cycle status, gene expressions, and transcriptomic profiles of the isolated CEnCs for SNEC-I therapy; and (3) to ascertain a realistic translational workflow from obtaining donor tissues, isolating the CEnCs using the SNEC-I harvesting approach and incorporating the process to determine the number of harvested CEnCs. This can demonstrate in vivo functionality with the prepared non-cultured CEnCs within the established rabbit model of bullous keratopathy using SNEC-I therapy.

## 2. Methods

### 2.1. Study Design

**Figure 1:**
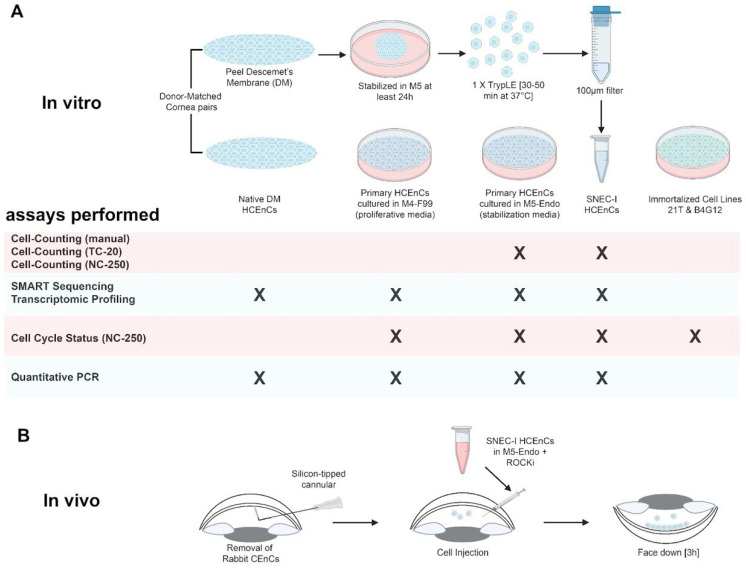
A diagram demonstrating the (**A**) in vitro and (**B**) in vivo experimental design and approach of this study. All experiments were performed with at least three biological repeats. Diagram created in Biorender.com.

This study was divided into two parts, consisting of (1) a series of experimental in vitro studies to evaluate and optimize the cell counting process and to analyze the cell cycle profiles, as well as (2) a functional in vivo component using an established rabbit model of bullous keratopathy (Figure 1). These studies were acknowledged by the local centralized institutional review board of SingHealth (Reference: 2015/2320) and were conducted in accordance with the Declaration of Helsinki. The in vitro experiments were designed to evaluate the accuracy and consistency of manual versus automated cell-counting approaches using propagated primary human CEnCs. Subsequently, the cell cycle status, gene expressions, and transcriptomic profiles of the SNEC-I-harvested primary human CEnCs in comparison to its donor-match native corneal endothelium and propagated CEnCs were evaluated. Immortalized corneal endothelial cell lines were also included in this analysis. All experiments were performed with a minimum of three paired donor corneas (Supplementary Table S1). For the in vivo functional studies, six New Zealand white rabbits (*Oryctolagus cuniculus*; 2.5–3.5 kg, 3–6 months old) were used. The use of these animals and their care and treatment strictly adhered to the regulation of the ARVO statement for the Use of Animals in Ophthalmic and Vision Research, and all experimental procedures were approved by the Institutional Animal Care and Use Committee of SingHealth, Singapore (2020/SHS/1544). This study was reported in accordance with ARRIVE guidelines (https://arriveguidelines.org, accessed on 19 May 2025) [25].

### 2.2. Materials

Ham’s F12, Medium 199, Human Endothelial-SFM, Dulbecco’s phosphate-buffered saline (dPBS), TrypLE^TM^ Select (TS), OptiMEM-I Reduced Serum Medium (OptiMEM-I), bovine pituitary extract, Dulbecco’s Modified Eagle Medium (DMEM), gentamicin, amphotericin B, penicillin and streptomycin, and human recombinant epidermal growth factor (EGF) were purchased from Life Technologies (Carlsbad, CA, USA). Collagen IV from human placenta, trypan blue (0.4%), and chondroitin sulfate were purchased from Sigma (St. Louis, MO, USA). Insulin/Transferrin/Selenium (ITS) were purchased from Corning (Corning, NY, USA), and ascorbic acid was purchased from Spectrum Chemical Mgf. Corp. (New Brunswick, NJ, USA). MACS^®^ SmartStrainers (100 μM), human recombinant basic fibroblast growth factor (bFGF), and rho-associated, coiled-coil protein kinase (ROCK) inhibitor Y-27632 were purchased from Miltenyi Biotec (Bergisch Gladbach, Germany). FNC coating mixture was obtained from Athena Enzyme Systems (Baltimore, MD, USA). Liberase TH was purchased from Roche (Mannhein, Germany). Bovine serum, EquaFetal^®^, to supplement the culture medium, was purchased from Atlas Biologicals (Fort Collins, CO, USA). Staining solutions (Solution 18, Solution 12) and associated buffers (Solution 10, Solution 11) for use with the NucleoCounter NC-250 system were obtained from Chemometec (Allerod, Denmark).

### 2.3. Source of Human CEnCs

#### 2.3.1. Research-Grade Human Corneo-Scleral Tissues

A total of 21 donor cornea pairs and 1 single donor cornea deemed unsuitable for corneal transplantation were procured for this study (Supplementary Table S1) from either the Lions Eye Institute for Transplant and Research (Tampa, FL, USA) or the Saving Sight (Kansas City, MO, USA), with informed consent obtained from the next of kin [24]. All donor corneoscleral rims were preserved and transported to Singapore Eye Research Institute (SERI) in Optisol-GS (Bausch & Lomb, Rochester, NY, USA) at 4 °C until they were processed.

#### 2.3.2. Human Corneal Endothelial Cell Lines

Two human corneal endothelial cell lines, the SV40-immortalized B4G12 cell line [26] and the telomerase-immortalized 21T cell line [27], were used in this study.

### 2.4. Primary Cell Isolation and Cell Culture

#### 2.4.1. Primary Corneal Endothelial Cell Expansion Using the Dual Media Culture System

The cell culture technique utilized in the initial and optimization steps of this study and transcriptomic profiling was similar to previous studies [21,28]. The primary human CEnCs were isolated and propagated using our previously published dual media culture approach to the second passage [28]. As per our established protocol, the isolated CEnCs were first incubated in the corneal endothelial stabilization medium (M5-Endo medium; Human Endothelial-SFM supplemented with 5% serum and 10 μm Y-27632) overnight to stabilize the CEnCs, followed by subsequent culture in the proliferative medium (M4-F99; Ham’s F12/M199, 5% serum, 20 μg/mL of ascorbic acid, 1× ITS, 10 ng/mL of bFGF, and 10 μm of Y-27632) to promote the proliferation of the attached CEnCs. Once the CEnCs reached 80–90% confluence, cells were re-exposed to M5-Endo for at least one day before being sub-cultured via single-cell dissociation using TS for further cell expansion. For transcriptomic profiling, selected donor-matched primary cells were collected at two specific phases for comparative study: one at its proliferative phase at the first passage when the CEnCs were at 50% confluence in M4-F99 medium, and the other at its stabilization phase at the second passage after the confluent CEnCs were maintained in M5-Endo for at least five days. All cultures were maintained within a humidified atmosphere at 37 °C and 5% CO_2_.

#### 2.4.2. Isolation of Human CEnCs Using the SNEC-I Harvesting Approach

The isolation of primary human CEnCs via the SNEC-harvesting approach was performed as described in our previous study [24]. Briefly, the Descemet membrane–corneal endothelium (DM-CE) complex was peeled and incubated in M5-Endo medium for at least 24 h. Following incubation in M5-Endo medium, the DM-CE complex was dissociated in 800 μL of TS for 30–50 min until the CEnCs rounded up individually. The resulting single cells were collected and filtered through a 100 μM cell strainer to remove any remnant DM fragments. An additional 200 μL of TS was added to the dissociated remnants, collected, and passed through the same cell strainer to gather any remaining single cells. At this stage, collected SNEC-I-harvested single-cell solution was ready for cell counting (see below) or prepared for quantitative PCR analysis.

For SNEC-I surgery, SNEC-I-harvested single cells were centrifuged and resuspended in an Eppendorf tube in a volume of 150 μL of M5-Endo containing 10 μM of a Rho-associated kinase inhibitor, Y-27632. The cellular solution was slowly transferred into a 1.0 mL syringe through a 30-gauge needle. Then, the SNEC-I procedure was completed within 2 h of the preparation.

#### 2.4.3. Cell Culture Technique for Corneal Endothelial Cell Lines

The two human corneal endothelial cell lines were cultured and propagated as follows: human CEnC-B4G12 cell line [26] was maintained in DMEM supplemented with 10% FBS, 1× penicillin/streptomycin, and 50 µg/mL of gentamicin. Culture media were refreshed every 2–3 days, and cells were passaged at 80–90% confluence. HCEnC-21T cell line [27] was maintained in OptiMEM-I Reduced Serum Medium, supplemented with 8% FBS, 1× penicillin/streptomycin, 50 µg/mL of gentamicin, 200 ng/mL of calcium chloride, 0.08% chondroitin sulfate, 100 µg/mL of bovine pituitary extract, and 5 ng/mL of EGF.

### 2.5. Cell Counting Experiment

Cell counting studies were performed using two approaches, comparing standard manual hemocytometry with two automated cell counters (available in our laboratory), specifically the TC20 automated cell counter (Bio-Rad Laboratories, Watford, UK) and NucleoCounter NC-250 automated cell counter (Chemometec, Allerod, Denmark). TC20 (Bio-Rad) was used for cell counting because it is cost-friendly and is commonly used in many labs, whereas NucleoCounter NC-250 was required for cell counting as well as cell cycle analysis (i.e., the second part of the study).

In order to delineate the accuracy and the lower limits of cell counting of the two automated cell counters, human CEnCs expanded to the second passage were dissociated and subsequently serially diluted into four concentrations, encompassing undiluted neat, 1:2 dilution, 1:4 dilution, and 1:8 dilution. The rationale for using diluted samples is to help reduce the overall cell loss during the cell counting step for SNEC-I therapy. Dissociated human CEnCs were resuspended in 2 mL before being separated in duplicates of equal volume and labeled Set A and Set B. Set A samples were used for both manual and automated cell counting (see below), whereas Set B samples were seeded onto 7 mm FNC-coated glass coverslips and maintained for a week in M5-Endo to obtain a high density seeding of human CEnCs. This enabled a donor-matched validation of the accuracy of both manual and automated cell counts. Morphometric analysis was subsequently performed as previously described [24].

For manual counting and the use of TC20 automated cell counter, each cell concentration was loaded at 10 µL per hemocytometer chamber or automated cell-counting chamber (BioRad), respectively, with trypan blue at a 1:1 volume ratio for analysis. For the NucleoCounter NC-250 system, cells were loaded at 10 µL per chamber of an NC-slide A8 with Solution 18 (staining reagent containing a mixture of acridine orange and DAPI) at a 1:20 volume ratio.

### 2.6. Cell Cycle Analysis Experiment

The cell cycle analysis was performed on the NucleoCounter NC-250 system using its two-step cell cycle analysis protocol as per manufacturer’s instructions. Briefly, this assay allowed the determination of the cell cycle phase by measuring the DNA content within permeabilized cells using fluorescent, DNA-selective stains. The analyzed cells were distributed into three populations: (1) G1 phase (one set of paired chromosomes per cell); (2) S phase (DNA synthesis phase, with varied amounts of DNA within the cells); and (3) G2/M phase (cell growth/mitotic phase, with two sets of paired chromosomes per cell). This assay was first performed on two immortalized cell lines, HCEnC-21T and HCEnC-B4G12. Cell samples were collected as a pellet, washed with PBS, and prepared for cell cycle analysis as per manufacturer’s instructions. Briefly, the cells were resuspended in Solution 10 (lysis buffer) and Solution 12 [10 µg/mL 4′,6-diamidino-2-phenylindole (DAPI)] for five minutes at 37 °C, and this was immediately followed by adding Solution 11 (stabilization buffer) before analysis using the NucleoCounter NC-250 system. After the optimization process, cell cycle analyses of various cell types, including both HCEnC-21T and HCEnC-B4G12 cell lines, non-cultured CEnCs, and SNEC-I harvesting method, as well as cultured CEnCs in either M4-F99 medium (proliferative phase) or M5-Endo medium (stabilized phase), were comparatively assessed and analyzed.

### 2.7. Quantitative PCR

To examine and compare the effect of the isolation and culture techniques on human CEnCs and human corneal endothelial lines, an array of 11 genes involved in cell cycle (covering the entire cell cycle from G1/S-phase to M phase) was chosen for analysis (Table 1). Four pairs of donor corneas were used, with one cornea from each pair used as control (i.e., primary human CEnC on DM) and the fellow cornea used for non-cultured CEnC isolated for SNEC-I therapy. Additionally, two M4-cultured human CEnCs and the two immortalized cell lines, HCEnC-21T and HCEnC-B4G12, were used as comparative groups. We selected M4-cultured CEnCs over M5-cultured CEnCs, as the former group was more likely to demonstrate differences in cell cycle genes when compared to SNEC-I-harvested CEnCs.

The total RNA was extracted from cell samples using the PureLink™ RNA Mini Kit (Thermo Fisher Scientific, Waltham, MA, USA). The total RNA was treated to remove DNase using on-column DNase treatment from PureLink™. cDNA conversion was performed using High-Capacity cDNA Reverse Transcription Kit from Applied Biosystems™(Waltham, MA, USA). Quantitative PCR (qPCR) was performed on Roche Lightcycler 480 (Basel, Switzerland) with LightCycler 480 SYBR Green I Master qPCR mastermix (Basel, Switzerland). The results were normalized against endogenous glyceraldehyde 3-phosphate dehydrogenase (GAPDH), and analysis was performed using comparative Ct method to determine relative fold changes.

### 2.8. Transcriptomic Profiling

In order to validate the cell cycle status of the SNEC-I-harvested human CEnCs in comparison to its donor-matched corneal endothelium, a series of donor-matched transcriptomic comparisons were carried out, where four pairs of donor corneas were procured and isolated. The donor-matched expanded cells at the proliferative stage at passage 1, as well as the stabilized phase in the subsequent passage 2 (see Section 2.4.1), were used as controls.

Due to the limited number of primary human CEnCs available from each donor pair, transcriptome profiling for this study was performed at the single-cell level with the aid of single-cell transcriptome amplification via signal-mediated amplification of RNA technology (SMART), followed by high-throughput sequencing for transcriptome analysis at the cellular level. Specifically, the smart-seq2 approach was used for this study, and samples were prepared and processed using BGI solution via DNBSEQ-G400 sequencing platform (BGI, Shenzhen, Guangdong, China). Briefly, four donor-matched cell populations were prepared at different stages of isolation and culture steps from four independent donors. Collected samples were immediately placed in a cell lysis buffer and stored at −80 °C until they were processed. Specifically, the four groups of samples include (i) corneal endothelium on DM, (ii) SNEC-I-harvested CEnCs, (iii) proliferating CEnCs at the first passage, and (iv) stabilized CEnCs at the second passage. Subsequently, each lysed cell sample was subjected to PCR amplification, and the amplified products were assessed for quality using the Agilent 2100 Bioanalyser (Agilent Technologies, Santa Clara, CA, USA). Transposase-based library construction was then carried out on qualified amplified cDNA to achieve high-throughput sequencing.

Following transcriptomic profiling, standard bioinformatics analysis was performed to provide an overview of differentially expressed genes (DEGs; |log_2_FC| ≥ 1, Q value ≤ 0.001) and was carried out using the Dr. Tom online system (http://biosys.bgi.com/#/report/login, accessed on 19 May 2025). Standard bioinformatic QC and analysis were performed as previously described. Briefly, the fastq data was processed for transcript abundance estimates using *Salmon* (v1.4), followed by pseudocount mapping using *Tximport (v1.26.1).* The gene count matrix was imported to DESeq2 (v1.38.3) for downstream analysis. The data was normalized using the *count* function to adjust for gene expression variations between samples, followed by rlog transformation and principal component analysis using the *plotPCA ()* function. For cell cycle score analysis, the *cyclone ()* function from the *scran* package was used to assign cell cycle score for the sample using a set of pre-trained cell cycle marker genes for human cells [29].

### 2.9. Animal Surgeries

A total of six New Zealand White rabbits (*n* = 6 eyes) were used in this study. Only the left eye of each rabbit was used in this study to ensure animal welfare and to account for the potential inter-eye variability. Three eyes received SNEC-I therapy, and the remaining three eyes served as the untreated control group. A sham injection control group was deemed unnecessary for this study, as it was already performed in our previous study. All SNEC-I surgical procedures were performed by JSM. These surgical procedures and follow-up evaluations were performed under general anesthesia achieved by intramuscular injections of 5 mg/kg of xylazine hydrochloride (Troy Laboratories, Glendenning, NSW, Australia) and 50 mg/kg of ketamine hydrochloride (Parnell Laboratories, Alexandria, NSW, Australia), along with topical application of 1% lignocaine hydrochloride (Pfizer Laboratories, New York, NY, USA).

### 2.10. Lens Extraction Surgeries

The crystalline lenses of rabbits were extracted as described [21]. Briefly, mydriasis was achieved by administration of 1% tropicamide (Alcon Laboratories, Fort Worth, TX, USA) and 2.5% phenylephrine hydrochloride (Alcon Laboratories) eye drops approximately 30 min before lens extraction surgery. A clear corneal incision was made with a 2.8 mm disposable keratome. A 5.0 mm diameter continuous curvilinear capsulotomy of the anterior capsule was created under viscoelastic material (Viscoat; Alcon Laboratories) instilled into the anterior chamber. Hydro-dissection was performed using a 27-gauge cannula. The lens was then aspirated and removed with a standard phacoemulsification procedure using the White Star phacoemulsification system (Abbott Medical Optics, Santa Ana, CA, USA). Subsequently, the corneal incisions were sutured with 10/0 nylon sutures, and the rabbits were left aphakic with an intact posterior capsule for at least one week before SNEC-I procedures.

### 2.11. SNEC-I Surgery

The refined surgical procedure for SNEC-I therapy was based on our previous study [24]. Briefly, prior to SNEC-I, an intravenous dose of heparin (500 units in 1.0 mL) was administered to the rabbits to reduce intraocular fibrin formation. Subsequently, an anterior chamber maintainer was placed to infuse balanced salt solution (BSS) containing additional heparin at 1 unit per ml. Next, a paracentesis was created with a diamond knife to accommodate the insertion of a soft 30-gauge silicone-tipped cannula (Cat. #: SP-125053, ASICO, Westmont, IL, USA) for the removal of the corneal endothelium layer of the rabbit whilst keeping the DM intact. It should be noted that removal of the rabbit’s endothelium was performed carefully from limbus to limbus. A continuous flow of irrigation with BSS ensured that scraped endothelial cells did not remain on the surface of the DM. A solution of trypan blue was injected intracamerally to aid in the assessment of the denudation step. Here, areas of DM devoid of CEs were stained blue, and any areas with residual CE stood out against the blue-stained DM. The scraping process was then repeated, specifically targeting these areas until the entire DM was stained blue, indicating that the endothelial cells were removed. Subsequently, 0.5 mL of 100 μg/mL carbochol (Miostat^®^, Alcon Laboratories) was injected to achieve intraoperative miosis. Both the paracentesis incision and the anterior chamber maintainer paracentesis sites were secured with 10/0 nylon interrupted sutures. This was followed by a 0.2 mL anti-inflammatory and anti-infective subconjunctival injection of a 1:1 mixture of 4 mg/mL of dexamethasone sodium phosphate (Hospira, Melbourne, VIC, Australia) and 40 mg/mL of gentamicin sulfate (Shin Poong Pharmaceutical, Seoul, Republic of Korea). Using a syringe attached to a 30 G cannula, approximately 0.2 mL of aqueous humour was removed to make the anterior chamber shallow. SNEC-I-harvested CEnCs suspended in M5-endo medium supplemented with ROCKi Y-27632 (10 μM) were then injected through a separately tunnelled track through a 30 G needle. Immediately following the injections of CEnCs, rabbits were placed in a manner that ensured the cornea was in a downward position to facilitate the attachment of the injected cells onto the posterior corneal surface. Here, the rabbits were maintained for a minimum of three hours under volatile anesthesia.

### 2.12. Post-Operative Care

Following SNEC-I procedures, all rabbits received a post-operative regime of topical prednisolone acetate 1% (Allergan Inc, Madison, NJ, USA) and topical antibiotic moxifloxacin hydrochloride 0.5% (Vigamox, Alcon Laboratories, Fort Worth, TX, USA) four times a day. An intramuscular injection of 1 mL/kg of dexamethasone sodium phosphate (Norbrook Laboratories, Northern Ireland, UK) was also administered once daily. This medication regimen was maintained until the end of the study. It should be noted that additional anti-inflammatory and anti-infective subconjunctival injections of a 1:1 mixture of 4 mg/mL of dexamethasone sodium phosphate and 40 mg/mL of gentamicin sulfate were administered on top of the daily topical regimen in cases of observed acute corneal rejection.

### 2.13. Corneal Imaging and Measurement of Intraocular Pressure

All corneal imaging and measurements of intraocular pressures (IOP) were performed prior to transplantation, as well as at the following post-operative time-points: day 1, day 4, week 1, week 2, week 3, and week 4. Slit–lamp photographs were taken with a Zoom Slit Lamp NS-2D (Righton, Tokyo, Japan), and corneal cross-sectional scans and measurements of corneal thickness were performed using an anterior segment optical coherence tomography system (AS-OCT; Optovue, Fremont, CA, USA). Three measurements were taken for the assessment of central corneal thickness (CCT): at the corneal centre (0.0 mm) and at 1 mm on either side of the center (+1.0 mm, and −1.0 mm); mean values were calculated. Measurements of IOP were performed using a calibrated tonometer (Tono-pen Avia Vet, Reichert Ophthalmic Instruments, Buffalo, NY, USA). In vivo confocal microscopic (IVCM) images were obtained using the Heidelberg Retina Tomography (HRT) 3 system combined with the Rostock Corneal Module (HRT3/RCM; Heidelberg Engineering, Heidelberg, Germany).

### 2.14. Analysis of Corneas

All rabbits were followed for 28 days post-surgery before being sacrificed under anesthesia with an overdose of intracardiac injection of 85 mg/kg of sodium pentobarbitone (Jurox, Rutherford, NSW, Australia).

### 2.15. Immunohistochemistry

For immunohistochemistry, excised corneal samples were embedded in frozen section compounds (Surgipath; Leica Microsystems, Nussloch, Germany) and stored at −80 °C until they were processed. Serial sections of 8 µm sections were cut using a HM525 NX cryostat (Thermo Fisher Scientific, Waltham, MA, USA) and collected on polylysine-coated glass slides (Thermo Fisher Scientific, Waltham, MA, USA). Samples were rinsed and blocked in 5% normal goat serum in PBS for 30 min at room temperature (RT). Subsequently, samples were incubated with the primary antibodies at RT for 2 hours or at 4 °C overnight. The primary antibody used was anti-human nuclei antibodies (1:50; Merck Millipore, Burlington, MA, USA). Samples were then labeled with an AlexaFluor 488 conjugated goat anti-mouse IgG secondary antibody (2.5 µg/mL, Thermo Fisher Scientific, Waltham, MA, USA), mounted in Vectashield containing DAPI (Vector Laboratories, Newark, CA, USA), and visualized using a Zeiss Axioplan 2 fluorescence microscope (Carl Zeiss, Oberkochen, Germany).

### 2.16. Statistical Analysis

Statistical analysis was performed using SPSS version 27.0 (IBM SPSS Statistics for Windows, Armonk, NY, USA). All continuous data were presented as mean ± standard deviation (SD). Comparison between control group and other comparative groups was performed using unpaired *t* test. To enable direct comparison of the cell counting accuracy with the undiluted samples, the dilution factor (including 1:2, 1:4, and 1:8 dilutions) of diluted samples was corrected through multiplication by two, four, and eight times, respectively. For the cell cycle gene expression analysis, the mean DEGs in each endothelial cell group were compared and normalized to the control group (i.e., primary human CEnCs on DM). *p*-value of <0.05 was considered statistically significant.

## 3. Results

### 3.1. Cell Counting Performance of Manual Hemocytometry and Automated Cell Counters

**Figure 2:**
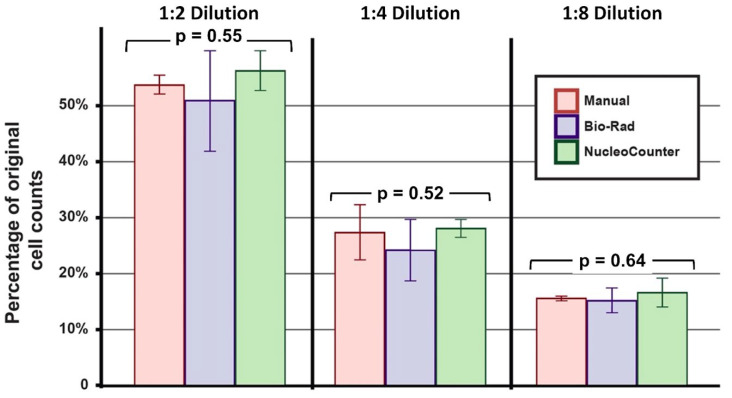
Performance of different cell counting approaches. The overall variance of manual cell counting using a hemocytometer, as well as automated cell counting using a Bio-Rad (TC20) and a NucleoCounter (NC-250), was assessed in a series of serial dilutions. Similar performance was observed among the three cell counting methods (all *p* > 0.05; one-way ANOVA test). All experiments were performed with three biological repeats (*n* = 3).

The human CEnCs cultured from three pairs of donor corneas were pooled separately for each donor and were analyzed using three different methods, namely, manual hemocytometry, Bio-Rad cell counter, and NucleoCounter. For undiluted samples, similar densities of CEnCs were measured with manual hemocytometry (1.04 ± 0.29 × 10^6^ cells/mL), a Bio-Rad cell counter (1.00 ± 0.32 × 10^6^ cells/mL), and a NucleoCounter (1.09 ± 0.24 × 10^6^ cells/mL; *p* = 0.92). Similarly, no significant difference was observed among the three methods for samples with 1:2 (*p* = 0.55), 1:4 (*p* = 0.52), and 1:8 dilutions (*p* = 0.64), suggesting that all three methods produced similar results up to 8× dilution (Figure 2).

#### Consistency of Cell Counting of Each Method for Cultured Human CEnCs

At a 1:2 dilution, the manual hemocytometry method was shown to have the least deviation (7.79 ± 3.30%) in cell count (compared to the undiluted sample), followed by the NucleoCounter (13.06 ± 6.85%) and Bio-Rad automated counter (14.52 ± 3.88%). At a 1:4 dilution (~2.5 × 10^5^ cells/mL), the NucleoCounter exhibited the best accuracy with the least deviation from the undiluted sample (12.49 ± 6.22%), followed by manual hemocytometry (14.58 ± 15.29%) and the Bio-Rad automated counter (17.53 ± 5.48%). At a 1:8 dilution, all methods demonstrated a considerable deviation greater than 20% from the undiluted samples (Table 2).

Based on non-cultured, freshly SNEC-I-harvested primary human CEnCs (*n =* 4 donor corneas), the effect of dilution on cell counting performance was similar to the results observed with the cultured CEnCs. NucleoCounter was shown to exhibit good cell counting accuracy at a 1:2 dilution (3.10 ± 2.77%) and 1:4 dilution (11.49 ± 15.36%), but not at a 1:8 dilution (33.34 ± 13.16%; Table 2).

### 3.2. Cell Cycle Analysis

Cell cycle analysis was first performed and optimized using two immortalized human corneal endothelial cell lines, namely, B4G12 and 21T cell lines, followed by cultured human CEnCs and SNEC-I, harvested human CEnCs. Performed in three technical replicates, both cell lines demonstrated considerably different cell cycle profiles, with B4G12 cell line having a substantially lower number of cells in G1 (57.40 ± 10.75% vs. 71.45 ± 0.64%; *p* = 0.21) and a higher number of cells in the S phase (20.05 ± 8.56% vs. 10.95 ± 1.48%; *p* = 0.28), G2/M phase (16.40 ± 1.98% vs. 6.45 ± 0.78%; *p* = 0.022), and sub-G1 phase (6.15 ± 0.21% vs. 11.15 ± 1.63%; *p* = 0.049) when compared to the 21T cell line (Table 3). With a starting density of two million cells for each cell line, both cell lines demonstrated a consistent cell cycle profile (within the same cell line) up to a 1:32 dilution (Supplementary Table S3), highlighting the validity and reproducibility of using NucleoCounter NC-250 (with a minimum of approximately 6.00 × 10^4^ cells/mL required for accurate analysis). Moreover, from a 1:64 dilution onwards, in-system warning messages appeared where low cell numbers were detected, which in turn may affect the generated analysis.

After the initial optimization, we examined the cell cycle profiles of donor-matched human primary CEnCs in two culture media conditions, including proliferative F99-M4 medium and its stabilized Endo-M5 medium, for at least one week. Human CEnCs stabilized in Endo-M5 medium exhibited a significantly higher number of cells in the G1 phase (92.86 ± 1.56% vs. 87.25 ± 3.77%; *p* = 0.042) and a lower number of cells in the S phase (0.87 ± 0.77% vs. 3.17 ± 1.37%; *p* = 0.035) and G2/M phase (3.73 ± 0.83% vs. 6.92 ± 2.01%; *p* = 0.033) when compared to human CEnCs in proliferative F99-M4 medium (Table 3). When compared to cultured human CEnCs, the majority of freshly isolated, human CEnCs via the SNEC-harvesting approach were locked in the G1 phase (94.19 ± 3.63%), with a small amount in the sub-G1 phase (5.20 ± 3.47%) and nearly none in the S (0.35 ± 0.23%) or G2/M phase (0.06 ± 0.07%**)**. Compared to both populations of CEnCs in either proliferative F99-M4 medium or stabilized Endo-M5 medium, the propagated CEnCs had a significantly higher proportion of cells in the G2/M phase when compared to the SNEC-I-harvested CEnCs (*p* < 0.005; Table 3).

### 3.3. Gene Expression Analysis

#### 3.3.1. G1/S Phase Genes

**Figure 3:**
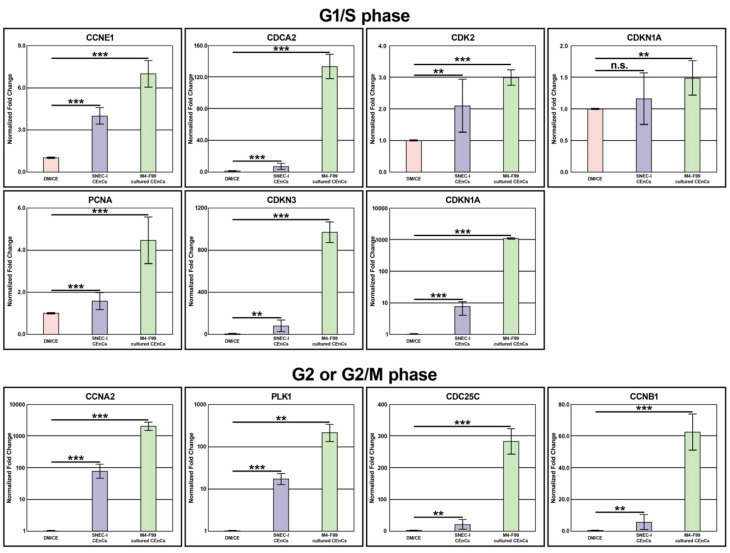
Cell cycle gene expressions. A graphic summary of the gene expression of 11 cell cycle genes involved in G1/S, G2, and G2/M phases of 3 different populations of human corneal endothelial cells (CEnCs), including primary CEnCs (DM/CE group), freshly isolated non-cultured CEnCs intended for simple non-cultured endothelial cell injection (SNEC-I group), and cultured CEnCs in M4 proliferative medium. Expressed values are normalized to the value of the control group, primary CEnC (the DM/CE group). The mean fold-change to DM/CE is presented as mean ± standard deviation (SD). Please note that the values in CCNA2, CDKN2A, CDKN3, and PLK1 groups are presented in log_10_ scale. Statistical comparison was between DM/CE, SNEC-I CEnC, and M4-F99 cultured CEnC groups (n.s. = non-significant; * *p* < 0.05, ** *p* < 0.01, *** *p* < 0.001; unpaired T-test). All experiments were performed with three biological repeats (*n* = 3).

Seven genes that were involved in the G1/S phase, including CCNE1, CDCA2, CDK2, CDKN1A CDKN2A, CDKN3, and PCNA, were examined. Using primary human CEnCs on DM as the reference group, the freshly harvested CEnCs exhibited considerably closer levels of gene expression across all seven genes than the proliferating CEnCs (Figure 3 and Supplementary Table S4). Gene expression analysis of the freshly isolated SNEC-I-harvested group showed six (85.7%) genes within 10-fold, and one (14.3%) between a 10-to-100-fold change from the native corneal endothelium. In contrast, cultured human CEnCs group proliferating in M4-F99 medium showed four (57.1%) genes within a 10-fold change, two (28.6%) genes between 100-to-1000-fold change, and one (14.3%) gene was more than a 1000-fold change compared to primary CEnCs.

#### 3.3.2. G2 or G2/M Phase Genes

Four genes that were involved in G2 or G2/M, including CCNA2, CCNB1, CDC25C, and PLK1, were examined. Similar to the above, the freshly harvested human CEnCs exhibited similar gene expression in all four genes of the native corneal endothelium when compared to cultured human CEnCs (Figure 3 and Supplementary Table S4). In the SNEC-I-harvested group, one (25.0%) gene was within 10-fold, and three (75.0%) genes were within a 10-to-100-fold change to primary human CEnCs. In contrast, the proliferating human CEnC group showed one (25.0%) gene within a 10-to-100-fold change, two (50.0%) genes within a 100-to-1000-fold change, and one (25.0%) gene with over a 1000-fold change from the native corneal endothelium. Within the cell line groups, all four (100.0%) genes expressed a >100-fold change when compared to primary human CEnCs, indicating their highly proliferative ability (Supplementary Table S4).

### 3.4. Transcriptomic Profiles of Cultured and Non-Cultured CEnCs

**Figure 4:**
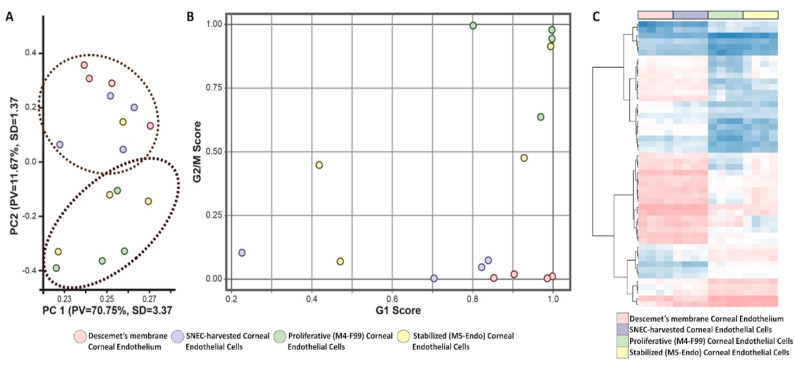
Transcriptomic profiling of human CEnCs. Transcriptomic analysis of the 4 populations of donor-matched primary CEnCs. (**A**) Principal component analysis (PCA) of donor-matched samples in the 4 conditions (*n* = 4 biological repeats). (**B**) Transcriptomic cell cycle analysis showing both G1 and G2/M scores of the donor-matched samples in the 4 conditions (*n* = 4 biological repeats). (**C**) Heat map analysis of the similarities and differences in the transcriptomic profiles among 4 groups of corneal endothelial cells, based on the top 50 differentially expressed genes. All experiments were performed with four biological repeats (*n* = 4).

To further elucidate the cell cycle status of the corneal endothelium and its SNEC-I-harvested counterpart, we performed transcriptomic analysis on four donor-matched cell populations using RNA-seq. The four populations of samples were the (i) DM with the native corneal endothelium, (ii) SNEC-I-harvested human CEnCs, (iii) proliferating human CEnCs in M4-F99 medium at the first passage, and (iv) human CEnCs in M5-Endo medium at the second passage stabilized for at least one week. Our results showed a total of 929 DEGs between the native corneal endothelium and SNEC-I-harvested CEnCs. A marked increase of 6666 DEGs and 3802 DEGs was detected between native CE compared to proliferating human CEnCs and stabilized human CEnCs, respectively. Principal component analysis was performed to assess the transcriptomic similarity between the human CEnC samples, with results showing the clustering of CEnCs on DM (Figure 4A; red) and SNEC-I-harvested CEnCs (Figure 4A; blue), indicating that at the transcriptome level, SNEC-I-harvested CEnCs are similar to the naïve corneal endothelium when compared to donor-matched expanded populations (Figure 4A; green and yellow). Notably, cell cycle analysis showed that both the corneal endothelium (Figure 4B; red) and its donor-matched SNEC-I-harvested CEnCs (Figure 4B; blue) have high G1 scores. CEnCs in proliferative media (Figure 4B; green) have higher G1 and G2/M scores (indicating a mixture of non-proliferative/stabilized and proliferative cells), whereas CEnCs in stabilization media (Figure 4B; yellow) have low G2/M scores (indicating lower mitotic activities). Similarly, heat map analysis of the transcriptomic profiles (based on the top 50 DEGs) demonstrated close resemblance between DM-CE cells and SNEC-I cells (Figure 4C). Altogether, these results supported the findings of the qPCR on cell cycle gene expression.

### 3.5. Pre-Clinical Functionality of SNEC-I Therapy

**Figure 5:**
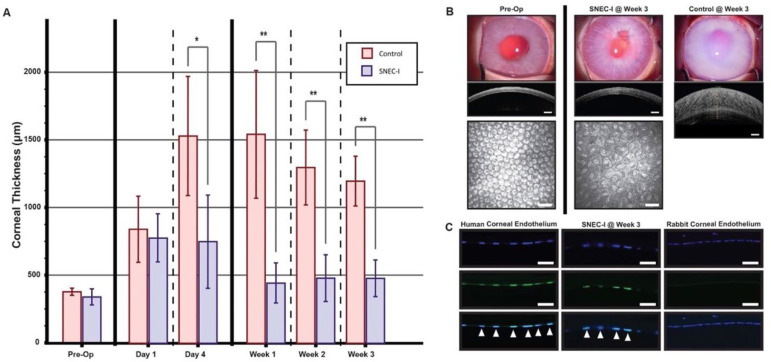
Functionality of SNEC-I in a rabbit model of bullous keratopathy. (**A**) Graph depicting the mean central corneal thickness in rabbits treated with SNEC-I (*n* = 3 rabbits; blue) compared to untreated controls (*n* = 3 rabbits; red) at the following time points: pre-operatively, day 1, day 4, week 1, week 2, and week 3 (* *p* < 0.05, ** *p* < 0.01). (**B**) Representative slit–lamp images and their corresponding anterior segment optical coherence tomography images of corneal thickness taken pre-operatively, as well as at week 3 for SNEC-I-treated and control rabbits (scale bar: 500 μm). In vivo confocal images are also included for both pre-operative and week 3 SNEC-I-treated rabbits (scale bar: 50 μm). (**C**) Representative immunofluorescent staining of DAPI (top row) and human-specific nuclear antibodies (middle row), with merged images (bottom row) showing co-localization (white arrow). Images include sections from human cadaveric donor corneas (left), excised corneas from rabbits receiving SNEC-I (center), and the contralateral rabbit cornea (right) (scale bar: 50 μm).

Using freshly SNEC-I cells, a series of cell counts were obtained from pairs of donor corneas (see Supplementary Table S2b). Following the SNEC-I procedure, the corneas of rabbits receiving SNEC-I therapy progressively cleared over the follow-up period, with clarity maintained throughout the study duration (Figure 5A,B). In contrast, corneas in the control group became edematous by day 4 (Figure 5A) and remained hazy throughout the follow-up period (Figure 5B; control, week 3). The IOP of all treated eyes remained within the normal range in this study. The mean CCT in the SNEC-I group initially increased to 783.7 μm ± 168.5 μm on day 1 but remained at 750.3 μm ± 338.4 μm on day 4. By week 1, the mean CCT significantly reduced to 450.7 μm ± 143.9 μm, with values of 484.4 μm ± 143.9 μm and 484.2 μm ± 130.1 μm observed at weeks 2 and 3, respectively (Figure 5A). In contrast, rabbits in the control group exhibited markedly higher CCTs (>1000 μm) from day 4 onward, and these values remained elevated for the rest of the follow-up period (Figure 5A; * *p* < 0.05 at day 4, ** *p* < 0.01 thereafter). Immunohistochemistry staining with a human-specific nuclei antibody confirmed these observations. Positive staining patterns for the human-specific nuclei antibody, co-localized with DAPI staining can be seen, as indicated by the white arrows on sections of cadaveric human donor cornea (Figure 5C, lower left panel). Similarly, co-localized staining was observed in the nuclei of the corneal endothelium in rabbits that received cells from SNEC-I (Figure 5B, lower middle panel). In contrast, no staining was detected in the corneal endothelium of rabbit cornea section (Figure 5C, lower right panel).

## 4. Discussion

With the current global shortage of donor corneas, innovative strategies are urgently required to tackle this unmet need [7]. Cell replacement therapy using primary CEnCs provides a potentially promising solution for the treatment of corneal endothelial diseases, though significant translational and regulatory barriers need to be overcome [23]. Previously, we demonstrated that SNEC-I may serve as a simple yet effective way of increasing the utilization of unsuitable donor corneas for treating corneal endothelial diseases [24]. In this work, using donor-matched samples, we demonstrated that the freshly isolated non-cultured human CEnCs were closer to the native corneal endothelium based on the analyses of cell cycle status, gene expressions, and transcriptomic profiles. We further described the entire process, streamlining it for clinical translation, in which a critical component of obtaining accurate numbers of isolated cells for SNEC-I therapy is included. More importantly, for the first time, we showed that 1.85 ± 0.23 × 10^5^ of injected CEnCs was sufficient to achieve functional recovery in the rabbit model of bullous keratopathy.

For conventional corneal transplantation, a required minimum corneal endothelial cell density of between 2200/mm^2^ and 2500/mm^2^ was established for donor corneas used in penetrating or endothelial keratoplasty, respectively [30,31,32]. In fact, studies have shown that higher pre-operative donor ECD has been associated with increased graft survival rate of endothelial keratoplasty [33,34], suggesting that the long-term success of corneal transplants is associated with pre-operative ECD of the donor graft tissue [11,30]. For SNEC-I therapy, we previously described a conjectured estimation of obtainable single-cell corneal endothelial yield based on the donor ECDs from specular microscopy imaging [24]. Therefore, establishing a rapid, reliable, and reproducible method for determining the yield of SNEC-I-harvested endothelial cells would be invaluable during the clinical translation of SNEC-I therapy. Currently, there exist several methods for cell counting, including manual hemocytometry, automated cell counting, and flow cytometry-based methods [35]. Manual hemocytometry is a simple method, but it can be time-consuming, operator-dependent, and associated with considerable variability [36,37]. This has prompted a shift toward automated cell counting for many different cell types in various research settings, including during GMP-compliant manufacturing processes [38]. In our study, we observed similar outcomes in the cell counts between manual hemocytometry and the two automated cell counters, highlighting the potential of automated cell counters in replacing the conventional manual hemocytometry method. The rapidity and ease of use of an automated cell counter can enhance workflow efficiency, thereby improving the overall cost-effectiveness of cell therapy.

As the outcome of the SNEC-I therapy is reliant on the overall cellular yield of the isolated human CEnCs, it is critical to streamline the process to reduce any possible “loss” of cells throughout the isolation-to-injection preparation, including the cell counting step. For the isolation of the DM, although donors of all ages can be procured for SNEC-I harvesting, an important consideration will be to dissect the DM-CE complex into smaller pieces during the peeling step, especially for DM from younger donors under 40 years. This is due to the scrolling of the peeled DM during the incubation period, as we observed in this study: isolated DM from younger donors had the tendency to form a tighter scroll, as reported [39,40,41].

In this study, we incorporated the use of NucleoCounter-250 as the primary tool for rapid automated cell counting for SNEC-I-harvested CEnCs. Our initial goal was to minimize the number of cells required for quantifying overall cell yield while ensuring reliable and reproducible results. This consideration was critical, as any use of isolated cells would reduce the final number of injectable cells available for the SNEC-I procedure. Our study showed that the cell counting accuracy of manual and automated methods was maintained at ~2.5 × 10^5^ cells/mL, with NucleoCounter-250 demonstrating the best performance. However, the reliability of all three methods declined significantly at an eightfold dilution (~1.3 × 10^5^ cells/mL), with inaccuracies ranging from 21% to 34%. This is likely due to the lowest limit of the machine’s performance for accurate counting. These findings highlight that the described dilution system relies on a higher cellular yield of SNEC-I-harvested CEnCs, ideally 3.0 × 10^5^ cells/mL, which was achievable from paired donor corneas with an ECD exceeding 2000 cells/mm^2^. For paired donors with lower corneal ECD or single-donor isolation, we propose an alternative approach that involves the adjustment of the volume of TrypLE solution used for dissociation to 1 mL. From this solution, 20 µL can be used for cell counting with the NucleoCounter NC-250. We also recommend maintaining a threshold of approximately 1.0 × 10^5^ cells/mL whenever possible when using the NucleoCounter NC-250 to ensure accurate results. Other automated cell counters could also be used for endothelial cell counting as long as the performance accuracy is validated against the reference standard (e.g., manual hemocytometry).

Translating cell-based therapy products, including human CEnC therapy, from bench to bedside calls for strict regulatory control and oversight. Many regulatory bodies, including those in Singapore, the US, and the European Union, adopt a risk-based approach in determining the level of scrutiny when it comes to evaluating cell-based therapy products [23]. Evaluation criteria include the intended use (homologous versus non-homologous), level of manipulation, any combination with other vehicles/devices, proliferative ability, long-term functionality, and pre-clinical/clinical data [23]. Therefore, developing a cell-based therapy product without the need for cellular expansion may overcome the regulatory barrier and shorten the bench-to-bedside translation of the therapy.

To better evaluate the differences in the isolated non-cultured human CEnCs (intended for SNEC-I therapy) when compared to primary human CEnCs on DM, we performed a combination of cell cycle status, genomic, and transcriptomic analyses. All results consistently supported the fact that SNEC-I cells are minimally manipulated, based on their close resemblance to the DM-CE cells. Human CEnCs are known to possess very limited in vivo proliferative ability in human eyes due to the cells being arrested in the quiescent non-proliferative G1 phase of the cell cycle [17,18,19]. We found that more than 99% of the SNEC-I-harvested human CEnCs were in the G1 (94.2%) or sub-G1 phase (5.2%), with only 0.4% in the S or G2/M phase. In contrast, M4- or M5-cultured human CEnC demonstrated that 10.1% and 4.6% of the cell population, respectively, were in the S or G2/M phase. It should be noted that our study used the NucleoCounter NC-250 system, which analyzed cell cycle status by measuring the DNA content within permeabilized cells using the fluorescent DAPI stain that binds to DNA. This is similar to the principle of using flow cytometry to measure the cell cycle status [42,43]. Although flow cytometry is most commonly used for analyzing cell cycle status, this approach is considerably more laborious from technical and analytic standpoints when compared to the NucleoCounter system. The validity and reliability of the NucleoCounter NC-250 results are supported by similar gene expression observations, where both cell lines (which are known to have high proliferative activity) demonstrated a high proportion of cells in the S or G2/M phase as opposed to uncultured human CEnCs (only 0.4% of the cell population in S or G2/M).

There are several advantages of the proposed SNEC-I therapy for treating corneal endothelial diseases. First, it can utilize donor corneas that are deemed unsuitable for conventional endothelial keratoplasty. Depending on the national guidelines set out by different countries, the current minimal threshold of ECD is generally set between 2200 and 2500 cells/mm^2^ for endothelial keratoplasty [30,31,32]. Based on the calculation of CEnCs required to replace the central 7–8 mm area of the diseased corneal endothelium (the amount of area that is commonly replaced by conventional endothelial keratoplasty), our previous study demonstrated that donor cornea pairs with as low 1250 cells/mm^2^ can potentially be utilized for SNEC-I therapy [24], significantly expanding the potential pool of “suitable” donor corneas. Donor corneas with even lower ECD may potentially be used if the diseased corneal endothelium is limited to a smaller area (e.g., mild bullous keratopathy/FECD that affects only a small area of the endothelium, localized endothelial dysfunction secondary to herpetic simplex keratitis, etc.). Similarly, donor corneas with higher ECD but below 2200–2500 cells/mm^2^ can potentially be used for more than one patient with limited/milder endothelial disease via the SNEC-I approach. Second, donor corneas with good ECD (e.g., >2500 cells/mm^2^) but with donor factors that may challenge the preparation of DMEK graft (e.g., young donor age [44] and diabetes [45]) may also be suitable for SNEC-I therapy to reduce the wastage of tissues during the DMEK graft preparation process, should the preparation of the DMEK graft result in irreparable damage to the tissue.

Unlike traditional cell-based therapies that rely on extensive cell culture and propagation, the whole process of SNEC-I, from the isolation and incubation of the DM to the dissociation of CEnCs, can be completed within 72 h. This streamlined process can potentially ease both regulatory and manufacturing challenges. It is noteworthy to mention that cell cycle status is not directly related to cell function; therefore, a proof-of-concept pre-clinical rabbit bullous keratopathy study was performed to validate the cell function. Building on our previous SNEC-I findings, which showed cellular functionality of injected SNEC-I-harvested CEnCs that reversed corneal blindness in the rabbit model of bullous keratopathy [24], we demonstrated, for the first time, that an injection of 1.85 ± 0.23 × 10^5^ CEnCs was able to achieve functional recovery in these rabbits, even after complete limbus-to-limbus corneal endothelial debridement. Although functional recovery was evident through reductions in corneal thickness compared to controls, IVCM images taken at week 3 revealed a relatively lower endothelial cell density. However, it is plausible to consider, for future clinical applications, that corneal endothelial debridement can be refined by selectively removing only a targeted region (visual axis) of the patient’s damaged corneal endothelium while keeping the DM intact. This, in turn, may enhance the stabilized cell density and the longevity of SNEC-I therapy, which we aim to investigate further in subsequent long-term animal studies. We would also consider a flat mount approach for immunohistochemical analysis of the treated corneas in future pre-clinical studies to better characterize the in vivo biological responses.

One of the limitations of this study is that we did not compare the performance of the cell cycle status analysis of NucleoCounter-250 with a commonly used technique (i.e., flow cytometry). However, the validity and reliability of NucleoCounter NC-250 results were supported by the results from the corneal endothelial cell lines, cultured CEnCs, and non-cultured CEnCs (for the SNEC-I therapy). The flow cytometry approach usually requires a higher volume of cells, which may lead to undesirable loss of cells after cell sorting. Future larger pre-clinical studies with longer-term follow-up in a second animal model (e.g., 3–6 months) will be performed to ascertain the long-term efficacy, safety, and viability of SNEC-I therapy (using slit–lamp examination, AS-OCT, and IVCM) before advancing to first-in-human clinical trials. We will also conduct experiments to evaluate and compare the risk of immunological rejection between the cultured CEnCs and SNEC-I cells.

## 5. Conclusions

Our study demonstrates that non-cultured CEnCs isolated for SNEC-I therapy are minimally manipulated, rendering SNEC-I therapy an attractive novel approach for treating corneal endothelial diseases, with potentially fewer regulatory hurdles. In addition, we highlight the potential clinical utility of NucleoCounter NC-250 for automated cell quantification and cell cycle analysis, which can help streamline the processing and quality assurance pipeline of corneal endothelial cell therapy within the laboratory and/or within eye bank settings. Future pre-clinical studies evaluating the efficacy and safety of SNEC-I therapy using a regulatory-compliant protocol will help advance SNEC-I therapy to the clinic.

## Figures and Tables

**Table 1:** Gene expression assays used for quantitative PCR (qPCR) to analyze the corneal endothelial cell cycle.

Gene Symbol	NCBI Gene Reference	Gene Name	Cell Cycle Phase Involved	Primer Sequence Used *
CCNE1	NM_001238.1	Cyclin E1	G1/S	F: AAGGTTTCAGGGTATCAGTGGTG R: GGCTTTCTTTGCTCGGGCTTTG
CDCA2	NM_152562.4	Cell division cycle associated 2	G1/S	F: GAGGCAGGAAAAGAGTCCGAGA R: CTCCGACGTTTGGAGGACAACA
CDK2	NM_001798.5	Cyclin dependent kinase 2	G1/S	F: ATGGATGCCTCTGCTCTCACTG R: CCCGATGAGAATGGCAGAAAGC
CDKN1A	NM_000389.5	Cyclin dependent kinase inhibitor 1A	G1/S	F: CAGACCAGCATGACAGATTTC R: TTAGGGCTTCCTCTTGGAGA,
CDKN2A	NM_000077.5	Cyclin dependent kinase inhibitor 2A	G1/S	F: AAGGTCCCTCAGACATCCC R: TGTAGGACCTTCGGTGACTG
CDKN3	NM_005192.4	Cyclin dependent kinase inhibitor 3	G1/S	F: ATGGAGGGACTCCTGACATAGC R: TCTCCCAAGTCCTCCATAGCAG
PCNA	NM_182649.2	Proliferating cell nuclear antigen	G1/S	F CAAGTAATGTCGATAAAGAGGAGG R: GTGTCACCGTTGAAGAGAGTGG
CCNA2	NM_001237.5	Cyclin A2	G2	F: CTCTACACAGTCACGGGACAAAG R: CTGTGGTGCTTTGAGGTAGGTC
CDC25C	NM_001790.5	Cell division cycle 25C	G2/M	F: AGAAGCCCATCGTCCCTTTGGA R: GCAGGATACTGGTTCAGAGACC
CCNB1	NM_001354845.2	Cyclin B1	G2/M	F: GACCTGTGTCAGGCTTTCTCTG R: GGTATTTTGGTCTGACTGCTTGC
PLK1	NM_005030.6	Polo like kinase 1	G2/M	F: GCACAGTGTCAATGCCTCCAAG R: GCCGTACTTGTCCGAATAGTCC
GAPDH	NM_002046.4	Glyceraldehyde-3 phosphate dehydrogenase	Housekeeping gene	F: AGCCACATCGCTCAGACAC R: GCCCAATACGACCAAATCC

* F = forward; R = reverse.

**Table 2:** The effect of various sample dilutions on cell counting accuracy of different cell counting methods, including manual hemocytometry, Bio-Rad TC20, and NucleoCounter NC-250 automated cell counters.

Methods	Deviation at 1:2 Dilution	Deviation at 1:4 Dilution	Deviation at 1:8 Dilution
Propagated primary human CEnCs (cultured)			
Manual	7.79% ± 3.30%	14.58% ± 15.29%	26.0% ± 2.00%
Bio-Rad	14.52% ± 3.88%	17.53% ± 5.48%	21.7% ± 16.90%
NucleoCounter	13.06% ± 6.85%	12.49% ± 6.22%	33.50% ± 20.10%
SNEC-I-harvested human CEnCs (non-cultured) *			
NucleoCounter	3.10% ± 2.77%	11.49% ± 15.36%	33.34% ± 13.16%

CEnCs = corneal endothelial cells. All experiments were performed with three biological repeats (*n =* 3). * See Supplementary Table S2 for the breakdown of the SNEC-I human CEnCs.

**Table 3:** Comparison of the cell cycle profiles for various human corneal endothelial cells (CEnCs).

Cell Cycle Status	SNEC-I-Harvested CEnCs	Proliferating CEnCs (M4 + Rock I)	Stabilized CEnCs (M5)	Cell Line B4G12	Cell Line 21T
G1	94.19 ± 3.63	87.25 ± 3.77	92.86 ± 1.56	57.40 ± 10.75 *	71.45 ± 0.64 **
S	0.35 ± 0.23	3.17 ± 1.37 *	0.87 ± 0.77	20.05 ± 8.56 *	10.95 ± 1.48 **
G2/M	0.06 ± 0.07	6.92 ± 2.01 **	3.73 ± 0.83 **	16.40 ± 1.98 **	6.45 ± 0.78 **
Sub-G1	5.20 ± 3.47	2.67 ± 0.61	2.54 ± 0.51	6.15 ± 0.21	11.15 ± 1.63

All values are presented as mean ± standard deviation (SD) %. All experiments were performed with three biological repeats (*n* = 3). Comparison was made between each group, using SNEC-I-harvested CEnC group as the reference group. * *p* < 0.05; ** *p* < 0.005.

## Data Availability

All data supporting the findings of this study are available within the paper and its Supplementary Information.

## Supplementary Materials

The following supporting information can be downloaded at: https://www.mdpi.com/article/10.3390/cells14130986/s1, Table S1: Donor information and number of donors for different stages of experiments. Table S2: (a) Validation of cell counts of harvested CEnCs performed using NucleoCounter NC-250 and (b) cell counts of harvested CEnCs used in the pre-clinical SNEC-I study obtained using NucleoCounter NC-250. Table S3: Summary of the cell cycle analysis of two human corneal endothelial cell lines, including B4G12 and 21T. Table S4: Summary of the expressions of 11 cell cycle-related genes of five different human corneal endothelial cell (CEnC) populations, including primary human CEnC on Descemet membrane (DM-CE group), freshly isolated non-cultured human CEnCs intended for simple non-cultured endothelial cell injection (SNEC-I group), M4-cultured human CEnCs, human CEnC-B4G12 cell line, and human CEnC-21T cell line.
