# Supplementary material for: Cell Counting and Cell Cycle Analysis of Simple Non-Cultured Endothelial Cell Injection (SNEC-I) Therapy: Characterization for Clinical Translation
Source: Cells. 2025 Jun 27;14(13):986. doi: 10.3390/cells14130986 (PMC12248661; doi:10.3390/cells14130986)
Supplement: Supplementary file 1 [file cells-14-00986-s001.zip › cells-3518062-supplementary.pdf]

Supplementary Materials

Cell Counting and Cell Cycle Analysis of Simple Non-Cultured Endothelial Cell Injection (SNEC-I) Therapy: Characterization for Clinical Translation

Darren S. J. Ting [1,2,3,4,5,†], Gary S. L. Peh [1,2,†], Dawn J. H. Neo [1], Xiao Yu Ng [1], Belinda Y. L. Tan [1], Raymond C. B. Wong [6], Hon Shing Ong [1,2] and Jodhbir S. Mehta [1,2,7,*]

Supplementary Table S1. Donor information and number of donors for different stages of experiments.

S/N	Donor	Age	Sex	Days to culture	Cell count (OS/OD)	Cause of Death	Cell counting				Cell cycle			
							Seeding / Establishing Density	SNEC-I Isolation and Validation	Dilution	Density Validation	SNEC-I isolation	Culture Comparisons	Q-PCR	Transcriptomics
**1**	**Paired**	**3**	**M**	**8**	**4896/4964**	**Anoxic encephalopathy**	▪		▪	▪				
2	Paired	7	M	10	3311/3509	Trauma	▪		▪	▪				
3	Paired	4	M	13	4013/4122	Drowning	▪		▪	▪				
4	Paired	72	M	15	2169/2119	End-stage liver disease		▪						
5	Paired	64	M	13	2630/2607	Anoxic brain injury		▪						
6	Paired	63	F	10	2078/1891	Respiratory		▪						
7	Single	61	M	13	NA /2802	Chronic obstructive pulmonary disease		▪						
8	Paired	53	M	7	2734/2843	Cancer					▪			
9	Paired	56	F	11	2845/2799	Metastatic colorectal cancer					▪			
10	Paired	53	F	8	1974/2211	Trauma					▪			
11	Paired	63	F	18	2876/2769	Cancer					▪			
12	Paired	66	M	14	1218/1561	Cardiac					▪			
13	Paired	16	M	14	3230/3002	Gunshot wound						▪		
14	Paired	27	F	15	3022/3146	Postpartum complications						▪		
15	Paired	35	M	8	2994/2844	Cancer						▪		▪
16	Paired	34	F	14	2947/2866	Overdose						▪		▪
17	Paired	57	F	13	2356/2452	Gastrointestinal bleed								▪
18	Paired	65	F	15	2074/2039	Bowel perforation								▪
19	Paired	19	M	12	2830/2847	Gunshot wound							▪	
20	Paired	48	F	14	2513/2674	Respiratory							▪	
21	Paired	55	F	14	1225/1314	Congestive heart failure							▪	
22	Paired	56	M	19	3279/3322	Acute cardiac event							▪	

Supplementary Table S2a. Validation cell counts of harvested CEnCs performed using Nucleocounter NC-250.

Donor	Age	Cell count (OS/OD)	Nucleocounter count (per ml)
Paired	72	2169/2119	$3.21 \pm 0.29 \times 10^5$
Paired	64	2630/2607	$3.14 \pm 0.01 \times 10^5$
Paired	63	2078/1891	$2.69 \pm 0.40 \times 10^5$
Single	61	NA /2802	$1.73 \pm 0.10 \times 10^5$

Supplementary Table S2b. Cell counts of harvested CEnCs used in the pre-clinical SNEC-I study obtained using Nucleocounter NC-250

Donor	Age	Cell count (OS/OD)	Nucleocounter count (per ml)
Paired	73	1821/1754	$1.85 \pm 0.23 \times 10^5$
Paired	61	2217/2222	$2.44 \pm 0.16 \times 10^5$
Paired	58	3145/2809	$3.33 \pm 0.57 \times 10^5$

Supplementary Table S3. Summary of the cell cycle analysis of two human corneal endothelial cell lines, including B4G12 and 21T.

Cell cycle	1:1 dilution	1:2 dilution	1:4 dilution	1:8 dilution	1:16 dilution	1:32 dilution
			**B4G12 cell line**			
**G1**	57.4 ± 10.8	56.4 ± 8.4	55.7 ± 9.8	54.4 ± 9.3	53.1 ± 8.3	52.6 ± 11.0
**S**	20.1 ± 8.6	19.3 ± 6.5	20.5 ± 8.8	21.2 ± 9.1	20.5 ± 4.8	20.7 ± 8.6
**G2/M**	16.4 ± 2.0	18.2 ± 0.6	17.5 ± 0.4	17.7 ± 2.8	20.4 ± 2.8	18.3 ± 0.1
**Sub-G1**	6.2 ± 0.2	6.3 ± 1.2	6.4 ± 1.3	6.8 ± 3.0	6.1 ± 0.7	8.5 ± 2.6
			**21T cell line**			
**G1**	71.5 ± 0.6	70.6 ± 0.6	70.6 ± 0.5	70.5 ± 1.4	71.1 ± 0.6	70.1 ± 2.1
**S**	11.0 ± 1.5	11.4 ± 2.3	12.3 ± 1.1	12.5 ± 2.0	11.6 ± 0.9	12.4 ± 3.8
**G2/M**	6.5 ± 0.8	7.2 ± 0.9	6.7 ± 1.2	7.4 ± 0.5	7.1 ± 0.3	7.5 ± 0.1
**Sub-G1**	11.2 ± 1.6	10.9 ± 2.6	10.1 ± 2.8	9.7 ± 1.1	10.3 ± 1.8	9.9 ± 1.6

Supplementary Table S4. Summary of the expressions of 11 cell cycle-related genes of five different human corneal endothelial cell (CEnC) populations, including primary human CEnC on Descemet membrane (DM-CE group), freshly isolated non-cultured human CEnCs intended for simple non-cultured endothelial cell injection (SNEC-I group), M4-cultured human CEnCs, human CEnC-B4G12 cell line, and human CEnC-21T cell line.

Gene	DM-CE	SNEC-I	P-value	M4	P-value	B4G12	P-value	21T	P-value
G1/S phase									
CCNE1	1.00	3.98 ± 0.60	<0.001	7.02 ± 0.94	<0.001	1.49 ± 0.11	0.075	12.31 ± 0.54	<0.001
CDCA2	1.00	6.76 ± 3.75	<0.001	130.5 ± 14.7	<0.001	171.2 ± 3.7	<0.001	453.8 ± 6.0	<0.001
CDK2	1.00	2.09 ± 0.84	0.003	2.99 ± 0.24	<0.001	7.13 ± 0.15	<0.001	9.02 ± 0.05	<0.001
CDKN1A	1.00	1.16 ± 0.41	0.34	1.49 ± 0.27	0.009	0.22 ± 0.01	<0.001	1.23 ± 0.01	0.004
CDKN2A	1.00	7.42 ± 3.44	<0.001	1077 ± 38	<0.001	23052 ± 302	<0.001	22267 ± 318	<0.001
CDKN3	1.00	84.61 ± 55.09	<0.001	968.0 ± 95.9	<0.001	5443 ± 93	<0.001	1945 ± 170	<0.001
PCNA	1.00	1.57 ± 0.40	0.003	4.44 ± 1.10	<0.001	25.79 ± 2.21	0.004	25.87 ± 0.17	<0.001
G2 phase									
CCNA2	1.00	75.48 ± 51.89	0.001	1970 ± 629	<0.001	4793 ± 83	<0.001	8520 ± 74	<0.001
G2/M phase									
CCNB1	1.00	6.09 ± 4.74	0.007	62.68 ± 11.32	<0.001	100.6 ± 5.4	<0.001	177.02 ± 3.20	<0.001
CDC25C	1.00	21.04 ± 16.65	0.004	284.5 ± 39.3	<0.001	961.1 ± 16.3	<0.001	3188 ± 48	<0.001
PLK1	1.00	17.27 ± 5.71	<0.001	214.4 ± 97.5	0.005	311.0 ± 76.1	0.029	596.5 ± 116.0	0.018

DM-CE = Descemet membrane-corneal endothelial cells

The mean fold-change to DM-CE are presented in mean ± standard deviation (SD). The values are expressed in normalized values using primary human CEnC (the DM-CE group) as the reference/control group. All experiments were performed with three biological repeats (n=3).
